# *In vitro *antiproliferative/cytotoxic activity on cancer cell lines of a cardanol and a cardol enriched from Thai *Apis mellifera *propolis

**DOI:** 10.1186/1472-6882-12-27

**Published:** 2012-03-30

**Authors:** Dungporn Teerasripreecha, Preecha Phuwapraisirisan, Songchan Puthong, Kiyoshi Kimura, Masayuki Okuyama, Haruhide Mori, Atsuo Kimura, Chanpen Chanchao

**Affiliations:** 1 Department of Biology, Faculty of Science, Chulalongkorn University, 254 Phayathai Road, Bangkok 10330, Thailand; 2 Department of Chemistry, Faculty of Science, Chulalongkorn University, 254 Phayathai Road, Bangkok, 10330, Thailand; 3 Institute of Biotechnology and Genetic Engineering, Chulalongkorn University, 254 Phayathai Road, Bangkok 10330, Thailand; 4 Honeybee Research Group, National Institute of Livestock and Grassland Science, Ibaraki 305-0901, Japan; 5 Division of Applied Bioscience, Graduate School of Agriculture, Hokkaido University, Sapporo 060-8589, Japan; 6 Department of Biology, Faculty of Science, Chulalongkorn University, 254 Phayathai Road, Bangkok 10330, Thailand

**Keywords:** Antiproliferative activity, *Apis mellifera*, Propolis, Cancer cell, Cardanol, Cardol

## Abstract

**Background:**

Propolis is a complex resinous honeybee product. It is reported to display diverse bioactivities, such as antimicrobial, anti-inflammatory and anti-tumor properties, which are mainly due to phenolic compounds, and especially flavonoids. The diversity of bioactive compounds depends on the geography and climate, since these factors affect the floral diversity. Here, *Apis mellifera *propolis from Nan province, Thailand, was evaluated for potential anti-cancer activity.

**Methods:**

Propolis was sequentially extracted with methanol, dichloromethane and hexane and the cytotoxic activity of each crude extract was assayed for antiproliferative/cytotoxic activity *in vitro *against five human cell lines derived from duet carcinoma (BT474), undifferentiated lung (Chaco), liver hepatoblastoma (Hep-G_2_), gastric carcinoma (KATO-III) and colon adenocarcinoma (SW620) cancers. The human foreskin fibroblast cell line (Hs27) was used as a non-transformed control. Those crude extracts that displayed antiproliferative/cytotoxic activity were then further fractionated by column chromatography using TLC-pattern and MTT-cytotoxicity bioassay guided selection of the fractions. The chemical structure of each enriched bioactive compound was analyzed by nuclear magnetic resonance and mass spectroscopy.

**Results:**

The crude hexane and dichloromethane extracts of propolis displayed antiproliferative/cytotoxic activities with IC_50 _values across the five cancer cell lines ranging from 41.3 to 52.4 μg/ml and from 43.8 to 53.5 μg/ml, respectively. Two main bioactive components were isolated, one cardanol and one cardol, with broadly similar *in vitro *antiproliferation/cytotoxicity IC_50 _values across the five cancer cell lines and the control Hs27 cell line, ranging from 10.8 to 29.3 μg/ml for the cardanol and < 3.13 to 5.97 μg/ml (6.82 - 13.0 μM) for the cardol. Moreover, both compounds induced cytotoxicity and cell death without DNA fragmentation in the cancer cells, but only an antiproliferation response in the control Hs27 cells However, these two compounds did not account for the net antiproliferation/cytotoxic activity of the crude extracts suggesting the existence of other potent compounds or synergistic interactions in the propolis extracts_._

**Conclusion:**

This is the first report that Thai *A. mellifera *propolis contains at least two potentially new compounds (a cardanol and a cardol) with potential anti-cancer bioactivity. Both could be alternative antiproliferative agents for future development as anti-cancer drugs.

## Background

Propolis is a sticky resin produced by various bee species and is mainly derived from the resins collected by bees from the buds and barks of trees [1]. It is used for the construction and repair of hives [2] and is considered to act as a protective barrier against contaminating microorganisms [3]. Propolis from various geographical locations, bee species and seasons, as well as their extracts, have been reported to exhibit a diverse array of bioactivities, such as antibacterial [4], antifungal [5], antiparasitic [6], free radical scavenging [7], anti-inflammatory [8] and antiproliferative [9] activities. Due to the broad range of bioactivities ascribed to propolis, it has long been used in traditional medicine [10]. Furthermore, at present, propolis is deemed to be acceptable for use in foods, such as beverages, health foods and nutritional supplements, as well as in cosmetics and personal hygiene products like toothpaste and soap.

Propolis typically consists of resin and balsam (50%), wax (30%), oil (10%), pollen (5%) and other (5%) minor components [11]. The main bioactive chemical compounds in propolis are reported to be phenolic acid, terpenes, cinnamic acid, caffeic acid, several esters and flavonoids, the last of which includes flavones, flavanones, flavonols, dihydroflavonols and chalcones [12,13]. However, the chemical composition of propolis is qualitatively and quantitatively variable, depending on the available floral diversity at the location, the bee species and the season of collection [14,15]. Because the diverse array and types of chemical components in propolis vary in size and polarity, the solvents used to extract the propolis play a key role in the bioactivities, including anti-cancer activities, that are obtained in the crude extracts or subsequent fractions [16], due to the differential fractionation of components between different extracting solvents. In addition to organic solvents, edible vegetable oils, triglycerides and fatty acids have been used to extract propolis [9]. Given that bioactivity guided fractionation processes are commonly used to meet the logistic demands of enriching such a complex mixture of components, it is important to note that different cell lines have been reported to vary in their sensitivity to each of the different bioactive compounds isolated from propolis. Regardless, caffeic acid phenethyl ester (CAPE) currently seems to be the most interesting component isolated from propolis and is currently being developed as a potential anti-cancer drug since it can inhibit the *in vitro *growth of many cell lines [17] including the estrogen receptor positive (ER^+^) and negative (ER^-^) MCF7 and MDA231 cell lines, respectively [18], along with the chemoresistant PANC-1 cell line [19]. The mechanism of how CAPE inhibits the growth of cancer cell lines has been widely studied. In addition, CAPE has been reported to only be cytotoxic to cancer cell lines and not to normal cells *in vitro *[20,21], and this is additionally supported by the results from the systemic *in vivo *administration of CAPE [22]. Other than CAPE, artepillin C from Brazilian green propolis was reported to almost completely suppress the growth of human neurofibromatosis tumor xenografts in mice by blocking the oncogenic PAK1 signaling pathway [23]. Furthermore, the oil extract of Brazilian propolis, of which the significant bioactive compound is artepillin C, could effectively inhibit sarcoma 180 ascites tumor cells in male Swiss mice [9].

In contrast to Western medicine, traditional folklore based Eastern medicine is generally based upon the use of extracts from natural sources that consist of multiple components. Although their effects are not acute or their side effect(s) can be delayed, their chronic usage can result in the gradual accumulation of toxic compounds [24]. For example, with respect to propolis it has been shown that two caffeic acid esters in poplar propolis, prenyl caffeate isomers and phenylethyl caffeate, can act as allergens and sensitize individuals [25]. Thus, minimizing the allergen content in propolis or its extracts is important [26]. In contrast, although pure chemicals are used in Western medicine, which then avoids this type of problem along with antagonistic or undesired (non-intended) side affects, their effects are acute and side effects, especially the selection for chemoresistant cancers and antibiotic-resistant bacteria, are still highly problematical. Thus, it is important to find new classes of agents, such as those with different target sites or modes of action, in order to relieve this problem.

In this research, we aimed to isolate compounds with anti-proliferative/cytotoxic activities against human cancer cells from *A. mellifera *propolis collected from within the Nan province in Northern Thailand. Propolis was extracted sequentially with three solvents of decreasing polarity, and the crude extracts screened for antiproliferative/cytotoxic activity against five human cancer cell lines using the 3- (4, 5-dimethyl-thiazol-2-yl) 2, 5-diphenyl-tetrazolium bromide (MTT) assay. The crude propolis extract that displayed significant antiproliferative/cytotoxic activity was then further fractionated by column chromatography, using thin layer chromatography (TLC) pattern profiling and MTT bioassay guided selection of the fractions. The apparently pure bioactive fractions were then characterized for their formula structure by nuclear magnetic resonance (NMR) and electrospray ionization mass spectrometry (EIS-MS), whilst their *in vitro *cytotoxicity against the five human cancer cell lines was evaluated in comparison to a non-transformed (normal) human cell line using the MTT assay and assaying the cell morphology in tissue culture and DNA fragmentation pattern.

## Methods

### Propolis collection

Propolis of *Apis mellifera *was collected from an apiary in Pua district, Nan province, Thailand, during January 28 - February 1, 2010. It was kept in the dark by wrapping with aluminium foil until used.

### Bioassay-guided isolation (partition)

The extraction procedure essentially followed that reported by Umthong et al. [27] and Najafi et al. [28]. Propolis (90 g) was stirred with 400 ml of 80% (v/v) methanol (MeOH) at 100 rpm, 15°C for 18 h and then clarified by centrifugation at 7,000 rpm, 20°C for 15 min. The extract (supernatant) was harvested and the solvent removed by low pressure evaporation to leave the crude MeOH extract of propolis (CME). The residual propolis (pellet) was then sequentially extracted in the same way with 400 ml of dichloromethane (CH_2_Cl_2_) followed by hexane to yield the crude CH_2_Cl_2 _extract (CDE) and crude hexane extract (CHE), respectively. All three crude extracts were kept in the dark at -20°C until they were tested for their antiproliferation/cytotoxicity activity by the MTT assay.

### Chromatography

#### Quick column chromatography

A sintered glass (250 ml) column (0.063 - 0.2 mm in size, Merck) was tightly packed with silica gel 60 G using a vacuum pump. The crude propolis extract (CHE, CDE or CME) was mixed with silica gel 60 to a paste, left to dry and then sprinkled onto the packed column followed by a piece of filter paper (110 mm in Ø) and a cotton plug. The column was then eluted with a stepwise mobile phase of 1.5 L of each of 0:1, 1:3, 1:1, 3:1 and 1:0 (v/v) CH_2_Cl_2_: hexane, followed by 3:7 (v/v) MeOH: CH_2_Cl_2_, collecting 500 ml fractions. The purity of each fraction was determined by TLC (described below), and fractions with the same TLC profile pattern were pooled prior to solvent removal by low pressure evaporation. Fractions were then screened for antiproliferation/cytotoxic activity using the MTT assay as detailed below.

#### Adsorption chromatography

A silica gel 60 (90 g) column (250 ml) in hexane was prepared as described above. Fractions which showed a good antiproliferation/cytotoxic activity were dissolved in the appropriate solvent, mixed with silica gel 60 (5-7 g) and left at room temperature (RT) until dry. They were then transferred to the column and eluted as above except the stepwise elution gradient was comprised of 500 ml of 0:1, 1:1 and 1:0 (v/v) CH_2_Cl_2_: hexane and finally MeOH, and 2.5 ml fractions were collected. Fractions were screened for component composition by TLC profile patterns, with those with similar TLC profiles being pooled and then screened for antiproliferative/cytotoxic activity using the MTT assay.

#### Thin layer chromatography (TLC)

TLC plates (a silica coated plate, Merck) were cut to 5 × 5 cm^2 ^and each sample was loaded by a capillary tube onto five replicate plates. One of each of the five replicate plates was then resolved in a mobile phase of one of 0:1, 1:1, 3:1 and 1:0 (v/v) CH_2_Cl_2_: hexane or 1:19 (v/v) MeOH: CH_2_Cl_2_, respectively. After the mobile phase solvent permeated to the top line of the TLC plate, the TLC plate was removed, left at RT to dry and then the resolved compounds were visualized and location marked under ultraviolet light.

### Antiproliferation and cytotoxicity assays against human cancer cell lines

#### Transformed (cancer) and non-transformed cell lines

The five selected cancer cell lines used in this research were derived from human duet carcinoma (BT474, ATCC No. HTB 20), undifferentiated lung (Chaco I, National Cancer Institute), liver hepatoblastoma (Hep-G_2_, ATCC No. HB8065), gastric carcinoma (KATO-III, ATCC No. HTB 103) and human colon adenocarcinoma (SW620, ATCC No. CCL 227) cancers. In addition, the non-transformed human foreskin fibroblast cell line (Hs27, ATCC No. CRL 1634) was used as a comparative control. All cell lines were obtained from the Institute of Biotechnology and Genetic Engineering, Chulalongkorn University. The five cancer cell lines were cultured in RPMI 1640 medium containing 5% (v/v) fetal calf serum (FCS), while the Hs27 cell line was cultured in Basal Iscove medium containing 5% (v/v) FCS, at 37°C with 5% (v/v) CO_2 _[28].

#### Cell counts

Cells were removed from their culture flask using standard trypsin treatment until dislodged with gentle aspiration into single cell suspensions and resuspended to ten-fold the initial volume, or as appropriate, to allow counting on an improved Neubauer counting chamber. Cells positioning at four large corner squares of the hematocytometer were counted and so the number of cells was calculated as:

$$\begin{array}{c} \text{Concentration}\;\text{of}\;\text{cells}\;\text{(cells/ml)}\;\text{ = }\;\text{(Number}\;\text{of}\;\text{cells/4)}\;\times \;\text{dilution}\;\text{factor}\;\times \;\text{1}{\text{0}}^{\text{4}}\;\text{cells/ml}\\ \left[3\;-\;\left(4,\;5\;-\;dimethyl\;-\;thiazol\;-\;2\;-\;yl\right)\;2,\;5\;-\;diphenyl\;-\;tetrazolium\;bromide\right]\;\left(MTT\right)\;assay \end{array}$$

The MTT assay was performed as reported by Santos et al. [29] and Hernandez et al. [17]. For each of the five cancer cell lines, 5 × 10^3 ^cells in 200 μl of RPMI 1640 medium containing 5% (v/v) FCS were transferred per well of a 96 well tissue culture plate, and incubated at 37°C in 5% (v/v) CO_2 _for 24 h prior to the addition of 2 μl/well of the test extract in dimethylsulfoxide (DMSO) at various final concentrations. The addition of 2 μl/well of DMSO alone was used as the control. Cells were then incubated as above for 72 h before 10 μl of 5 mg/ml MTT was added and incubated for another 4 h. The supernatant was then removed, the cells permeabilized and the formazan crystals dissolved by aspiration in 150 μl of DMSO and 25 μl of 0.1 M glycine prior to measuring the absorbance at 540 nm by a microplate reader. Three replications of each trial were performed. By assuming an equal mitochondrial metabolic activity per living cell, the absorbance is then related to the relative number of viable cells and so is reduced, relative to the control, by any antiproliferation and/or cytotoxic activity of the test compound.

#### Estimation of the inhibition concentration at 50% (IC_50_)

The absorbance at 540 nm of the test compound treated cancer cells and the solvent only control was used to calculate the relative number of viable cells, setting that for the control as 100%. The relative number of viable cells, as a % of the control, was then calculated as follows:

$$\text{The}\;\text{relative}\;\text{(\% )}\;\text{number}\;\text{of}\;\text{viable}\;\text{cells}\;\text{ = }\;\frac{\text{(Abs}\;\text{of}\;\text{sample)}\;\times \;\text{100}}{\text{(Abs}\;\text{of}\;\text{control)}}$$

where (Abs of sample) and (Abs of control) are defined as the absorbance at 540 nm of the treated cells and the control cells, respectively.

The IC_50 _values were graphically obtained by plotting the absorbance obtained against the corresponding different concentrations of the test compound used, and are reported as the mean ± 1 standard error (SE). Data were statistically analyzed using the Kruskal-Wallis One Way Analysis of Variance. Significance was accepted at the *P *< 0.05 level.

### Chemical structure analysis by spectroscopy

#### Nuclear magnetic resonance (NMR)

To analyze the enriched bioactive compounds, 2-3 mg of each purified active fraction was dissolved in 500 μl of deuterated chloroform (CDCl_3_) and transferred into an NMR tube. The sample was analyzed and recorded by a Varian Mercury^+ ^400 NMR spectrometer operating at 400 MHz for ^1^H and 2D NMR (COSY, HSQC, HMBC) and 100 MHz for ^13 ^C nuclei in order to search for functional groups. The chemical shift in δ (ppm) was assigned with reference to the signal from the residual protons in the deuterated solvent and TMS was used as an internal standard.

#### Mass spectroscopy (MS)

For each purified fraction a 1-2 mg aliquot was dissolved in ethyl acetate (1 ml) and was then commercially analyzed at the National Science and Technology Development Agency (NSTDA, Thailand) using ESI-MS to evaluate the molecular weight and functional group composition.

### DNA fragmentation

The SW620 cancer cells or untransformed Hs27 cells (5 × 10^5 ^cells/flask/6 ml media) were cultured as above for 24 h and then exposed to the test fraction at the derived antiproliferation/cytotoxic IC_50 _concentration for 72 h, observing their morphology and cell number every 24 h. The morphology of the SW620 or Hs27 cells treated with each test compound was compared to those treated with only the DMSO solvent as the control. Cells were released by standard trypsin and aspiration, centrifugally washed at 2,000 × g at 15-25°C for 5 min and finally the cell pellet was resuspended in 200 μl of PBS. To this 20 μl of proteinase K (> 600 mAU/ml) was added and total DNA was extracted using a QIAMP mini kit (Qiagen, cat. no. 51304), as per the manufacturer's instructions. The extracted DNA was stored at -20°C until used, with the concentration and purity being evaluated by measuring the absorbance at 260 and 280 nm (A_260/280 _ratio of 2.0; and an A_260 _of 1 being equal to 50 μg/ml), and the appearance after electrophoretic resolution through a 1.8% (w/v) agarose-TBE gel, coresolving the samples with λ *Hin*dIII (1.25 μg) and 100 bp DNA ladder (0.5 μg) as DNA markers. After electrophoresis, the gel was stained with 10 μg/ml of ethidium bromide (EtBr) for 10 min, destained in distilled water for 20 min and the DNA visualized by ultraviolet transillumination.

## Results

### Crude extract of propolis from Apis mellifera

After sequential extraction of propolis with methanol, CH_2_Cl_2 _and hexane, the three crude extracts obtained (CME, CDE and CHE, respectively) varied in appearance, yield and antiproliferative/cytotoxic bioactivities (Table 1). Considering the order of the sequential extraction, that the highest yield by far was found in the last solvent extraction (CHE) means that it is likely to be a realistic reflection that most of the extractable propolis components were non-polar, although of course it should be noted that most of the propolis was not extracted in all three solvents. Nevertheless, the brown pigments in propolis are, therefore seemingly non-polar, whilst the viscous or sticky nature may represent the wax.

**Table 1:** The weight and character of crude *A.mellifera *propolis extracts from Nan, Thailand

Fraction	Weight (mg)	% of initial propolis	Character	Antiproliferative/cytotoxic
CHE	22,500	25.0%	Dark brown, sticky	Yes

CDE	1,320	1.47%	Yellow brown, sticky	Yes

CME	740	0.82%	Hazel	Weak (> 10 μg/ml)

### Antiproliferative/cytotoxic activity

#### Effect of CHE, CDE and CME on different cancer cell lines

**Figure 1:**
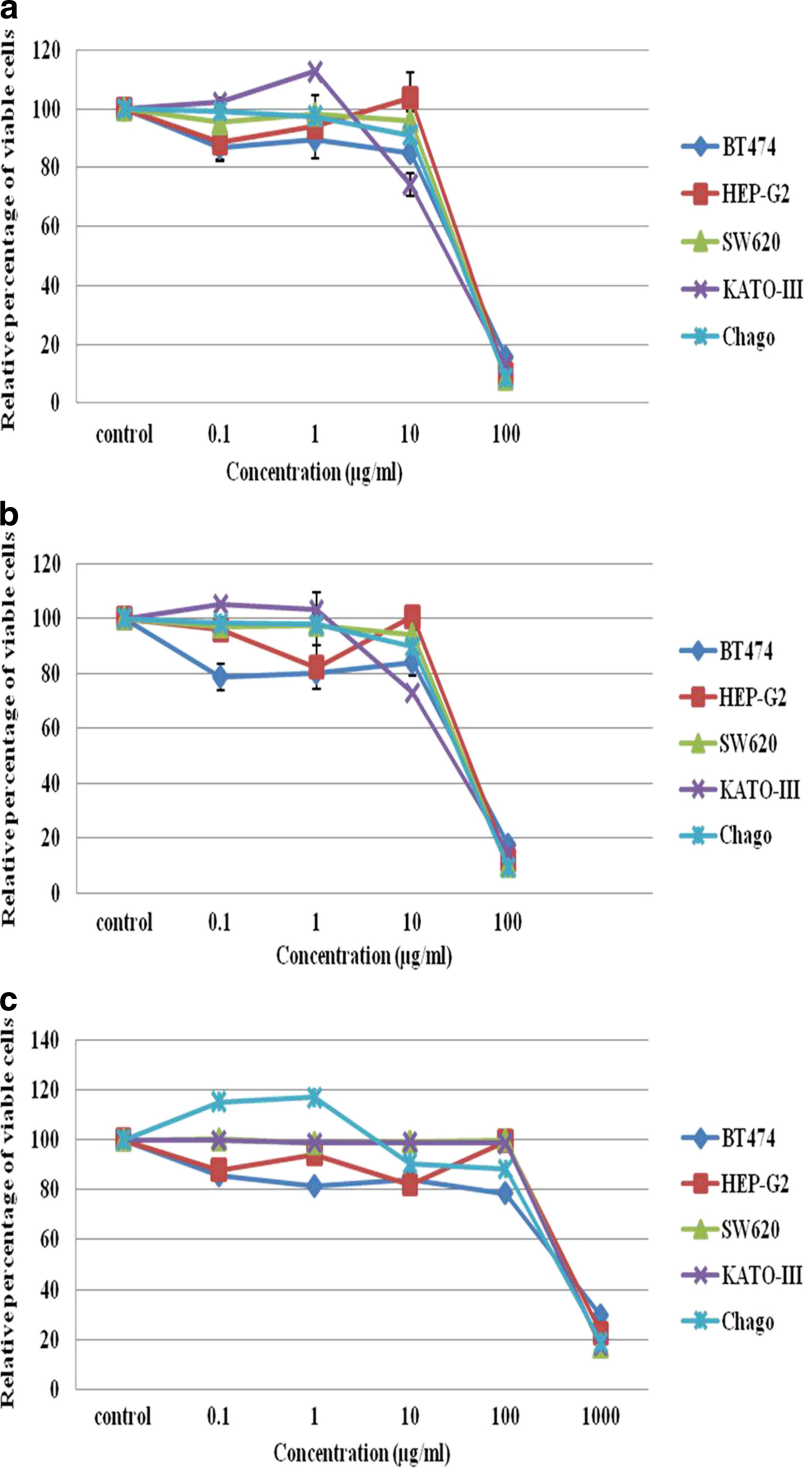
***In vitro *antiproliferative-cytotoxic activity of the (A) CHE, (B) CDE and (C) CME crude propolis extracts on five different human cancer cell lines after exposure to the test extracts for 72 h**. The data, as the percentage of viable cells relative to that of the control, are expressed as the mean ± 1 SE.

Five different cancer cell lines were used to screen for the *in vitro *antiproliferative/cytotoxic activity of the crude propolis extracts. Both the CHE and CDE revealed a strong and broadly similar antiproliferative/cytotoxic activity on all five cell lines in a dose-dependent manner (Figure 1).

In terms of the antiproliferative/cytotoxic IC_50 _values, the CHE and CDE were broadly numerically similar across all five cell lines and between both extracts, ranging from 41.3 μg/ml (CHE on Chaco) to 53.5 μg/ml (CDE on Hep-G2) (Table 2). In contrast, the CME was inactive at these concentrations showing a much weaker antiproliferative/cytotoxic activity (Figure 1) with over ten-fold higher IC_50 _values, ranging from 500 to 605 μg/ml (Table 2).

**Table 2:** The *in vitro *antiproliferative/cytotoxic IC_50 _values of the CHE, CDE and CME on selected cancer cell lines

Cancer cell lines	IC_50 _(μg/ml)		
	
	CHE	CDE	CME
BT474	48.3 ± 1.6^a^	52.6 ± 3.7^a^	500 ± 50^b^

Chaco	41.3 ± 3.75^a^	44.7 ± 0.33^a^	580 ± 20^b^

KATO-III	42.5 ± 6.61^a^	43.8 ± 6.5^a^	600 ± 50^b^

SW620	45.3 ± 0.33^a^	46.0 ± 0.57^a^	555 ± 7.5^b^

Hep-G2	52.4 ± 3.7^a^	53.5 ± 0.5^a^	605 ± 39.1^b^

Data are shown as the mean ± 1 SE and are derived from three independent repeats after a 72 h exposure to the test extracts. Means (within and between columns) with a different lowercase superscript letter are significantly different (*p *< 0.05; Kruskal-Wallis test)

#### Antiproliferative/cytotoxic effect of CHE fractions I - V on the different cancer cell lines

Although the CHE and CDE presented very similar antiproliferative/cytotoxic activities against the five selected cell lines, the yield of CHE was significantly (17-fold) greater and thus was selected for further fractionation by quick column chromatography. This yielded five fractions of distinct compositions, as determined by the TLC profile patterns, labeled as CHE fractions I - V, with by far the highest yield being found in Fraction V (4,300 mg), followed by fractions III and IV with a 13.4- and 15.9- fold lower yield, respectively, whilst fractions I and II were just minor components (Table 3).

**Table 3:** The yield and character of the five CHE fractions obtained after quick column chromatography

Fraction	Weight (mg)	Yield (% of CHE/total propolis)	Character	Antiproliferative/ cytotoxic activity^a^	TLC plates^b^
I	80	0.36%/0.09%	Clear wax	--	3

II	20	0.09%/0.02%	Clear yellow oil	--	3

III	320	1.42%/0.36%	Yellow oil	3 cell lines	1

IV	270	1.2%/0.30%	Yellow powder	2 cell lines	3

V	4,300	19.1%/4.78%	Dark brown oil	all 5 cell lines	2

^a^Number of the five human cancer cell lines in which a significant antiproliferative/cytotoxic activity was induced by the extract after a 72 h exposure, as determined by the MTT assay

^b^The minimum number of distinct compounds in the fraction as determined by evaluation of the different TLC plates

**Figure 2:**
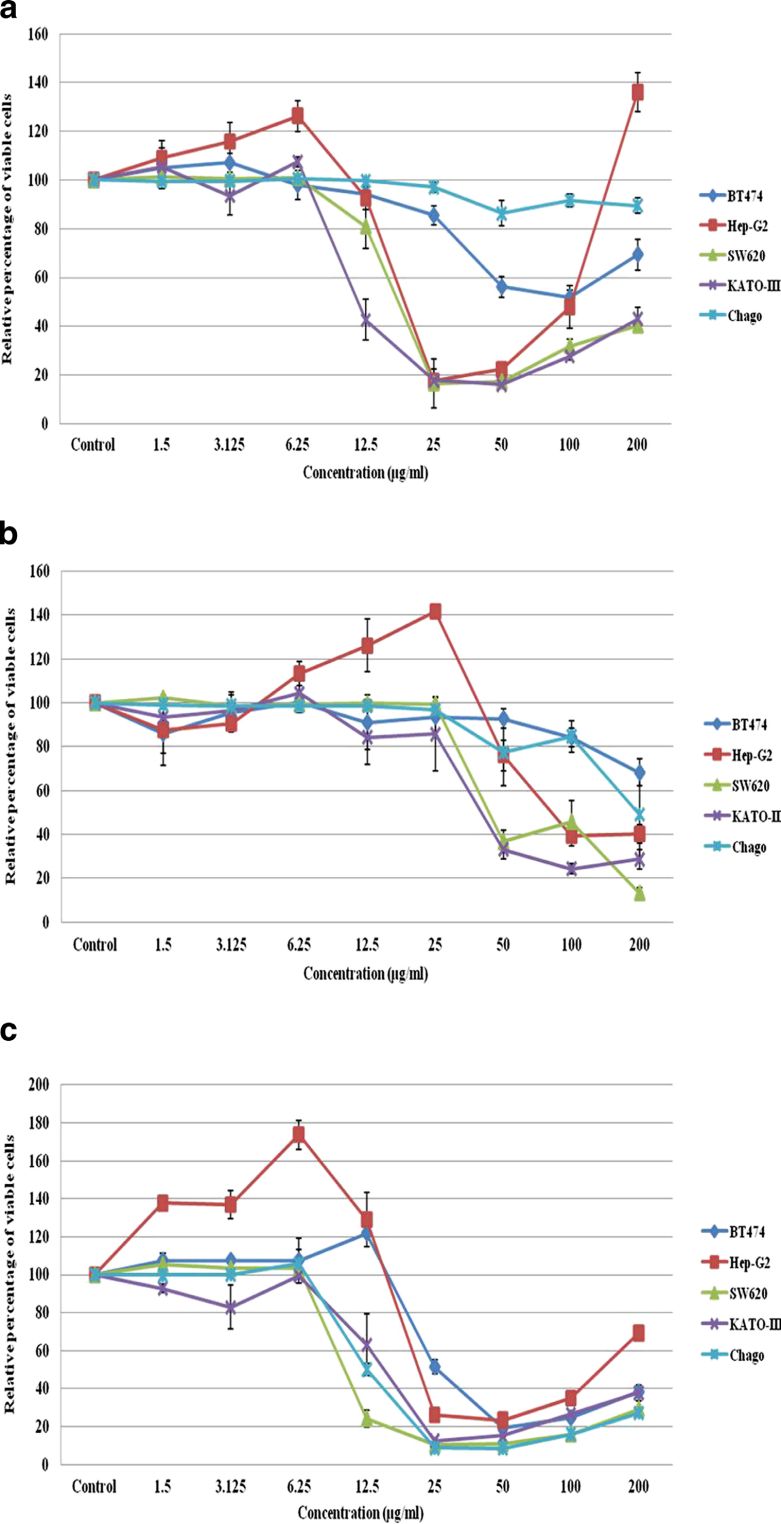
***In vitro *antiproliferative/cytotoxic activity of CHE fractions (A) III, (B) IV and (C) V on the five different cancer cell lines**. The antiproliferative/cytotoxic effect is expressed in terms of the percentage of viable cells relative to the control after 72 h exposure to the test fractions, and is shown as the mean ± 1 SE.

A strong *in vitro *antiproliferative/cytotoxic activity against all five selected human cancer cell lines was noted with fraction V, and against two and three of the cell lines for fractions IV and II, respectively (Table 4 and Figure 2), but no significant activity was noted for fractions I and II.

**Table 4:** The IC_50 _values for the *in vitro *antiproliferation/cytotoxic activity of CHE fractions I - IV on five human cancer cell lines

Cancer cell lines	IC_50 _values (μg/ml)				
	
	Fraction I	Fraction II	Fraction III	Fraction IV	Fraction V
BT474	ND	ND	ND	ND	29.36 ± 1.36

Chaco	ND	ND	ND	ND	12.75 ± 0.68

KATO-III	ND	ND	13.69 ± 1.44^a^	40.16 ± 2.66^b^	15.21 ± 2.13^a^

SW620	ND	ND	19.94 ± 1.83^b^	44.56 ± 1.89^c^	7.37 ± 0.23^a^

Hep-G_2_	ND	ND	19.37 ± 0.36	ND	22.22 ± 0.69

ND indicates no IC_50 _values were obtained since no significant antiproliferative/cytotoxic activity was found in this tested concentration range. Data is shown as the mean ± 1 SE from three independent repeats after a 72 h exposure to the test compound. Means (within and between columns) with a different lowercase superscript letter are significantly different (p ≤ 0.05; Kruskal-Wallis test)

Of the three positive fractions, fraction V had the highest antiproliferative/cytotoxic activity against each of the five selected cancer cell lines, with IC_50 _values ranging from 7.37 ± 0.23 μg/ml (SW260) to 29.36 ± 1.36 μg/ml (BT474). Fraction III showed broadly similar antiproliferative/cytotoxic activities, with IC_50 _values ranging from 13.69 ± 1.44 μg/ml (KATO-III) to 19.94 ± 1.83 μg/ml (SW620). Finally, fraction IV had the lowest antiproliferative/cytotoxic activity of the three positive fractions, and only on two of the five tested cell lines with IC_50 _values of 40.16 ± 2.66 μg/ml and 44.56 ± 1.89 μg/ml.

#### In vitro antiproliferative/cytotoxic effect of compounds 1 and 2 on the five different cancer cell lines

**Figure 3:**
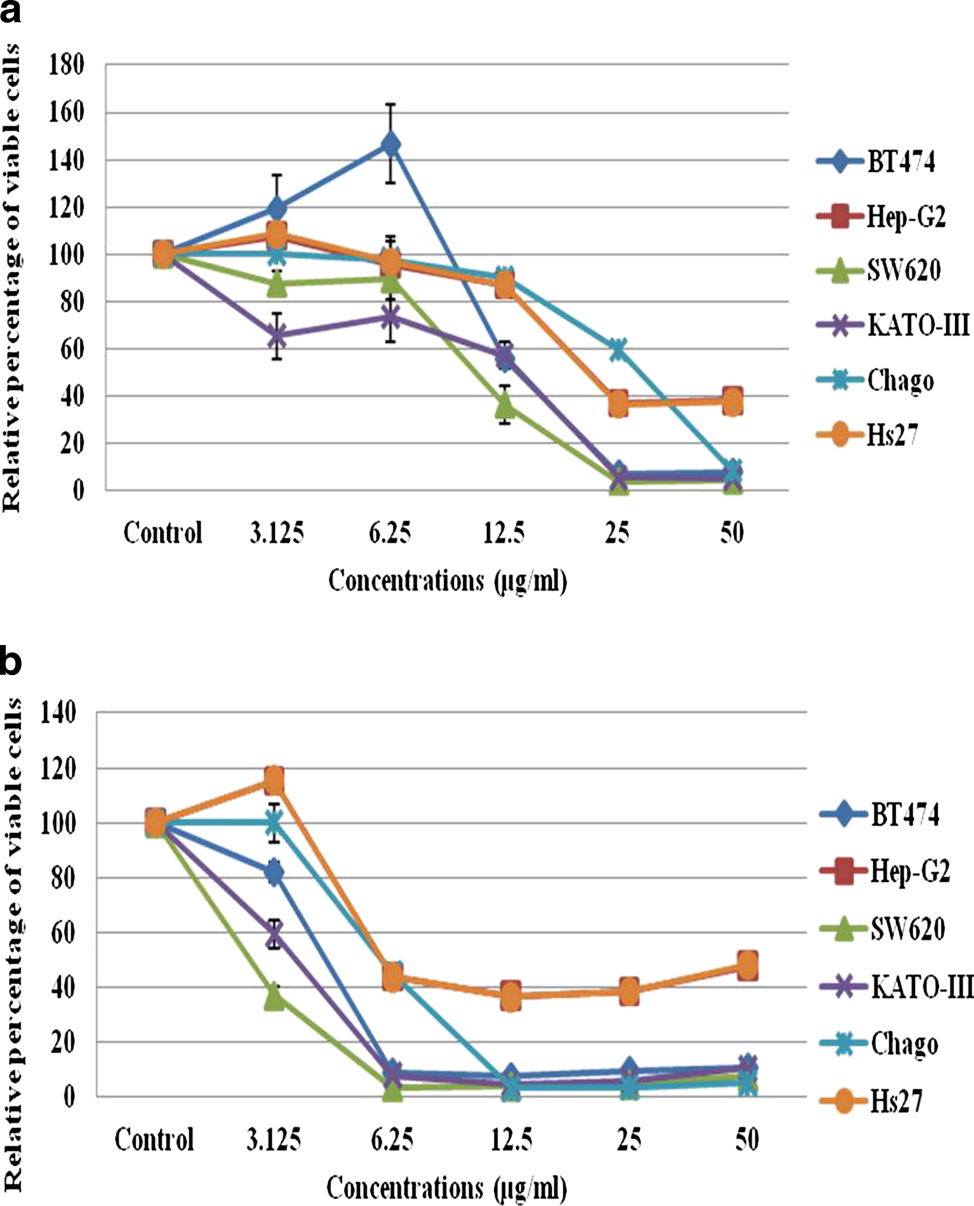
***In vitro *cytotoxic/antiproliferative activity of (A) compound 1 and (B) compound 2 on the five different cancer cell lines plus the non-transformed Hs27 cell line**. The antiproliferative/cytotoxic effect is expressed in terms of the percentage of viable cells relative to the control after 72 h exposure to the test compound, and is shown as the mean ± 1 SE.

Since CHE fractions V and III showed the highest antiproliferative/cytotoxic activities on the five screened human cancer cell lines, they were further purified by adsorption chromatography, yielding 88 and 92 fractions, respectively. However, in the TLC pattern profiles of all these fractions two dominant spots were clearly observed, one from CHE fraction III (compound 1) and the other from CHE fraction V (compound 2). After recovery from the TLC plates, compounds 1 and 2 were found to both have a strong antiproliferative/cytotoxic activity against the five different cancer cell lines in this MTT assay (Figure 3). The derived IC_50 _values of compound 1 for the SW620, KATO-III and BT474 cancer cell lines were 1.53- to 1.98- fold lower than that for the non-transformed Hs27 cell line, but in contrast, the IC_50 _values for the Hep-G_2 _and Chaco cancer cell lines were essentially the same as the Hs27 cell line (Table 5). Thus, the antiproliferation/cytotoxic activity of compound 1 on Hs27 is of concern.

**Table 5:** The *in vitro *antiproliferation/cytotoxic activity IC_50 _values of compounds 1 and 2

Cancer cell lines	IC_50 _value^1^		
	
	Compound 1 (μg/ml)^2^		Compound 2 (μg/ml/μM)
BT474	13.95 ± 0.9	4.41 ± 0.15	9.61 ± 0.33

Chaco	29.30 ± 1.08	5.78 ± 0.07	12.60 ± 0.15

KATO-III	13.71 ± 1.42	4.03 ± 0.13	8.78 ± 0.28

SW620	10.76 ± 0.92	< 3.125	< 6.81

Hep-G_2_	21.53 ± 0.35	5.97 ± 0.15	1.30 ± 0.33

Hs27	21.35 ± 0.52	5.97 ± 0.15	1.30 ± 0.33

^1 ^Data are shown as the mean ± 1 SE after a 72 h exposure to the test compounds, and are derived from three independent experiments

^2 ^The molar concentration of compound 1 could not be given as its molecular formulae, and thus molar mass, is not known

Compound 2 had a higher antiproliferative/cytotoxic activity than compound 1 for all five different cancer cell lines (Figure 3), with IC_50 _values ranging from < 3.13 to 6.0 μg/ml (~6.82 to 13.1 μM) for the five different cell lines, but it was equally effective against the non-cancer Hs27 cell line (Table 5), which is again of some concern for any potential *in vivo *application.

### Structure analysis of compounds 1 and 2

**Figure 4:**
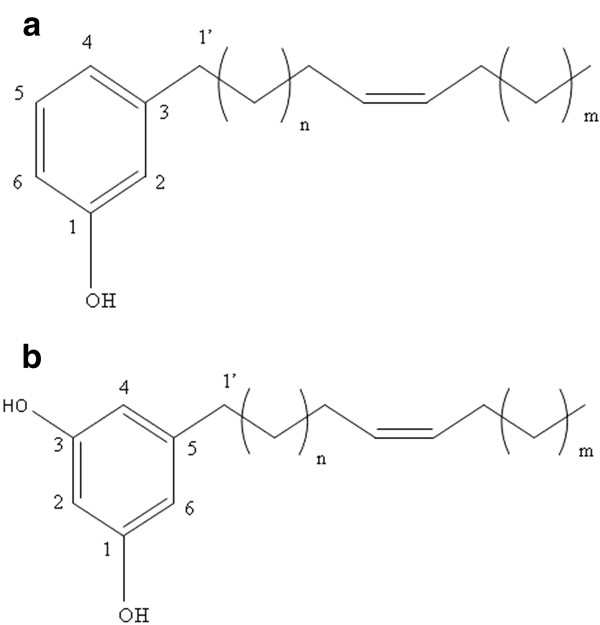
**The proposed formula structure of (A) compound 1, a cardanol and (B) compound 2, a cardol**.

Compounds 1 and 2 were analyzed by [^1^H]-NMR and ESI-MS spectroscopy. Compound 1 showed the characteristic signals of an m-disubstituted benzene [δ_H _7.05 (1H, H-5), 6.67 (1H, H-6), 6.58 (1H, H-2), 6.57 (1H, H-4)] and the characteristic resonances of the hydroxyl group from the chemical shift of carbon at δ_C _155.4 ppm. In addition, resonances at δ_H _5.28 (2H, m) suggested the presence of an olefinic proton. The Z-geometry of two olefinic protons, which were located at alkyl side chain, was assigned from the chemical shift of allylic carbons (δ_C _27.2 and 26.9). The presence of an alkyl group (R-) was indicated by the signal of methylenes (-CH_2_-) in the range of 1.2-2.5 ppm in addition to the terminal methyl group [0.82 (3H, t, *J *= 6.8 Hz)]. The chain length could not be determined exactly due to the lack of a calculated molecular mass, leaving an incompletely deduced structural formula, but it was categorized as a member of the cardanol group (Figure 4A).

The molecular formula of compound 2 was revealed to be C_31_H_54_O_2 _by ESI-MS [*m/z *(M + H)^+^], along with the characteristic signals of a m-trisubstituted benzene [δ_H _6.17 (2H, H-4, and H-6), 6.10 (1H, H-2)], and the characteristic resonances of the hydroxyl group from the single chemical shift of carbon at δ_C _156.5 ppm due to the symmetry. In addition, the resonances at δ_H _5.28 (2H, m) suggested the presence of olefinic protons. The Z-geometry of two olefinic protons, which were located at the alkyl side chain, was assigned from the chemical shift of allylic carbons (δ_C _27.2 and 26.9). The presence of the alkyl group (R-) was indicated by the signal of methylenes (-CH_2_-) in the range of 1.1-2.6 ppm in addition to thermal methyl [0.82 (3H, t, *J *= 6.8 Hz)]. From the NMR and ESI-MS results compound 2 was ascribed to be a member of the cardol group, although its exact formula is unresolved (Figure 4B).

### Morphology of the SW620 and Hs27 cells after in vitro exposure to compound 1 (cardanol) or compound 2 (cardol)

#### SW620 cancer cell line

**Figure 5:**
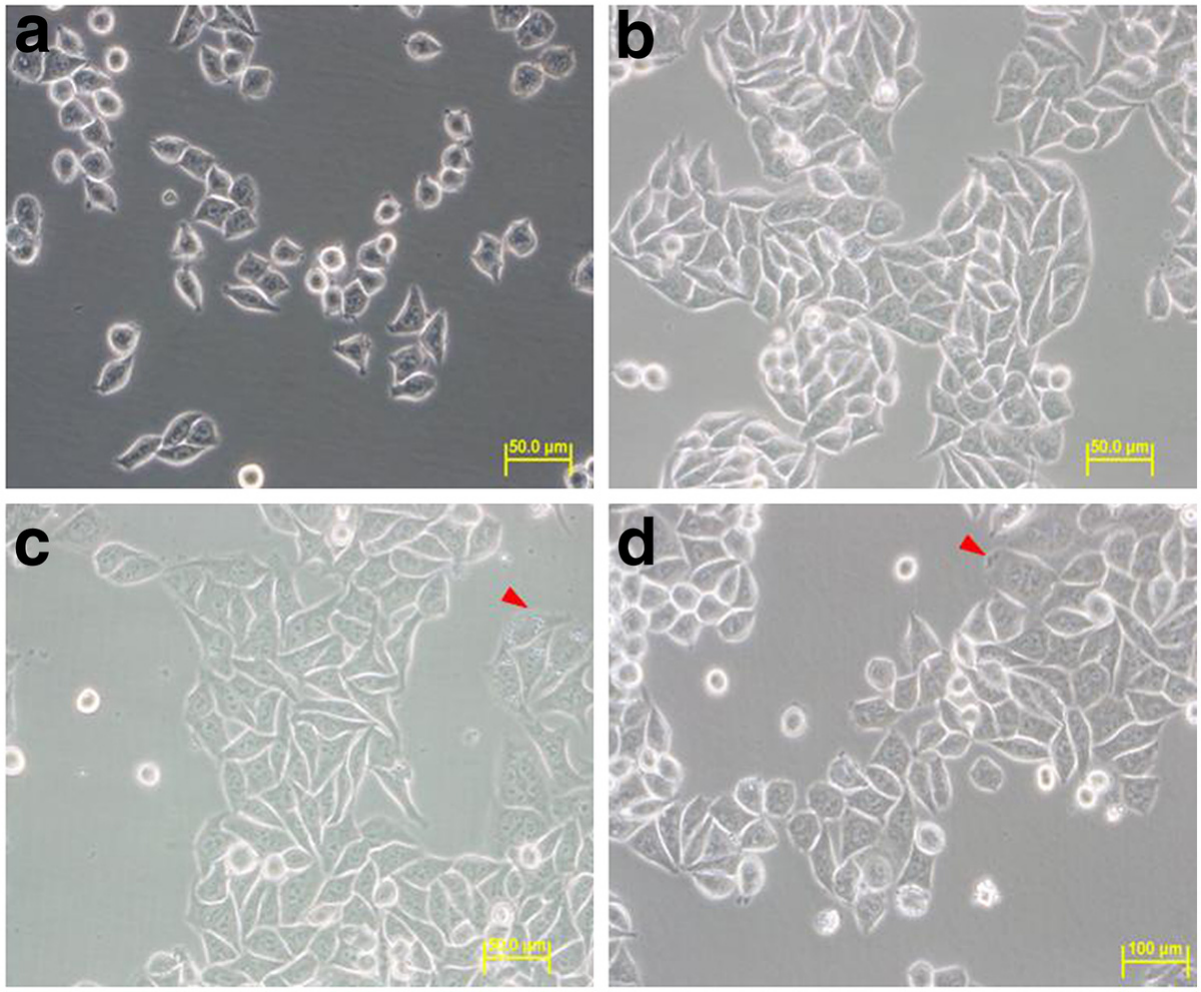
**SW620 cells after (A) 0 h culture and (B-D) after 48 h of culture with (B) the DMSO solvent alone (control) or with (C) compound 1 (cardanol) at its IC_50 _value (10.76 μg/ml), or (D) compound 2 (cardol) at its IC_50 _value (3.0 μg/ml; 6.54 μM)**. All images are magnified at 40×. Images shown are representative of at least five such fields of view per sample and three independent trials.

SW620 cells were cultured for up to 96 h in complete medium supplemented with DMSO alone (control) or the same amount of DMSO with either compound 1 (cardanol) or compound 2 (cardol) at their derived IC_50 _values for evaluation of their antiproliferation/cytotoxic activity, namely at 10.76 and 3.0 μg/ml, respectively. This is equivalent to 6.54 μM for compound 2, but the molarity of compound 1 is unknown since its molecular mass was not obtained. The cell morphology and cell number were observed at 0, 24, 48, 72 and 96 h. As set up (0 h), the cells looked flat and spindle shaped (Figure 5A). No significant change in the cell morphology was observed in all samples, that is the solvent only control and the cardanol and cardol treated cells, after 24 h of treatment time with cells still appearing flat and in a spindle shape (data not shown). However, after 48 h of *in vitro *culture vacuolation could be seen inside the cells treated with compound 1 or 2, but not in the control cells which were still normal (Figure 5).

**Figure 6:**
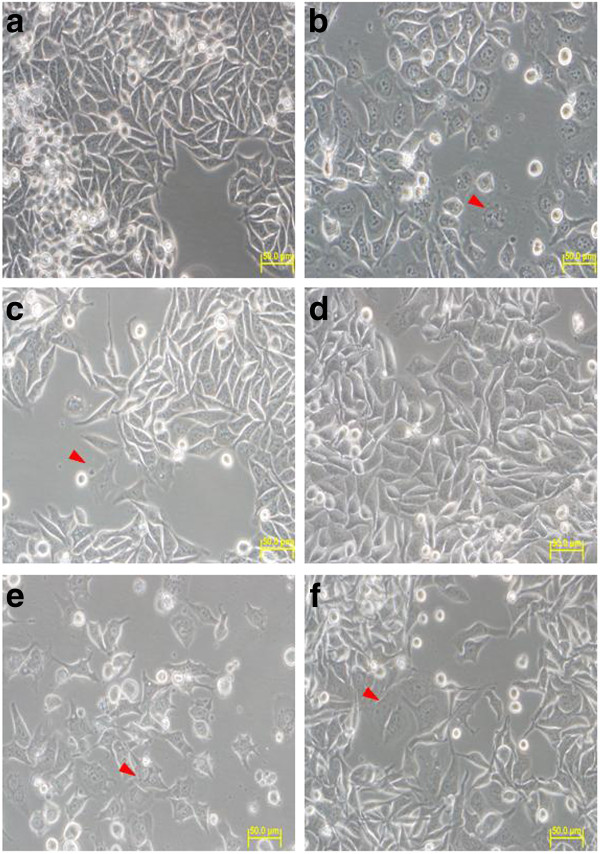
**SW620 cells after (A-C) 72 h or (D-F) 96 h of culture in (A, D) the DMSO solvent alone (control) or (B, E) with compound 1 (cardanol) at its IC_50 _value (10.76 μg/ml), or (C, F) compound 2 (cardol) at its IC_50 _value (3.0 μg/ml; 6.54 μM)**. All images are magnified at 40×. Images shown are representative of at least five such fields of view per sample and three independent trials.

By 72 h of cell culture, the control cells still appeared normal (but more dense and approaching or reaching confluency), whilst apparent DNA condensation within the nucleus was visible in both the cardanol and cardol treated cells (Figure 6). In addition, morphological changes and cell debris (indicated by a red arrow) were visible, as well as a reduced cell density compared to the control.

Finally, after 96 h of cell culture, whilst no change in the morphology of the control cells was noted, significantly higher levels of cells with DNA condensation within their nucleus (red arrow) along with cell debris, a loss of cell adhesion and a significantly reduced cell number were clearly visible in the cardanol and cardol treated cells (Figure 6).

#### Hs27 cells

**Figure 7:**
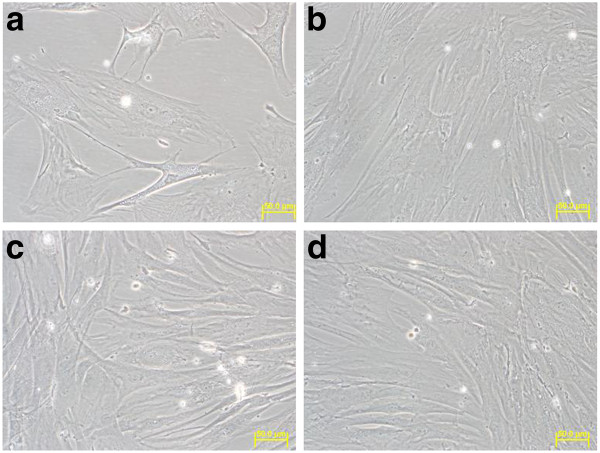
**The shape of Hs27 cells at (A) 0 h and (B-D) 96 h of *in vitro *culture with (A, B) DMSO solvent only (control), (C) compound 1 (cardanol) at its IC_50 _value (10.76 μg/ml)and (D) compound 2 (cardol) at its IC_50 _value (3.0 μg/ml; 6.54 μM)**. All images were magnified at 40×. Images shown are representative of at least five such fields of view per sample and three independent trials.

In contrast to that observed for the SW620 cancer cell line, no morphological changes were observed in the non-transformed Hs27 cell line after similar *in vitro *treatment with the same doses of cardanol or cardol (Figure 7). That is the cells looked flat and were attached to the substratum at all time points in all three treatments.

### DNA Fragmentation

**Figure 8:**
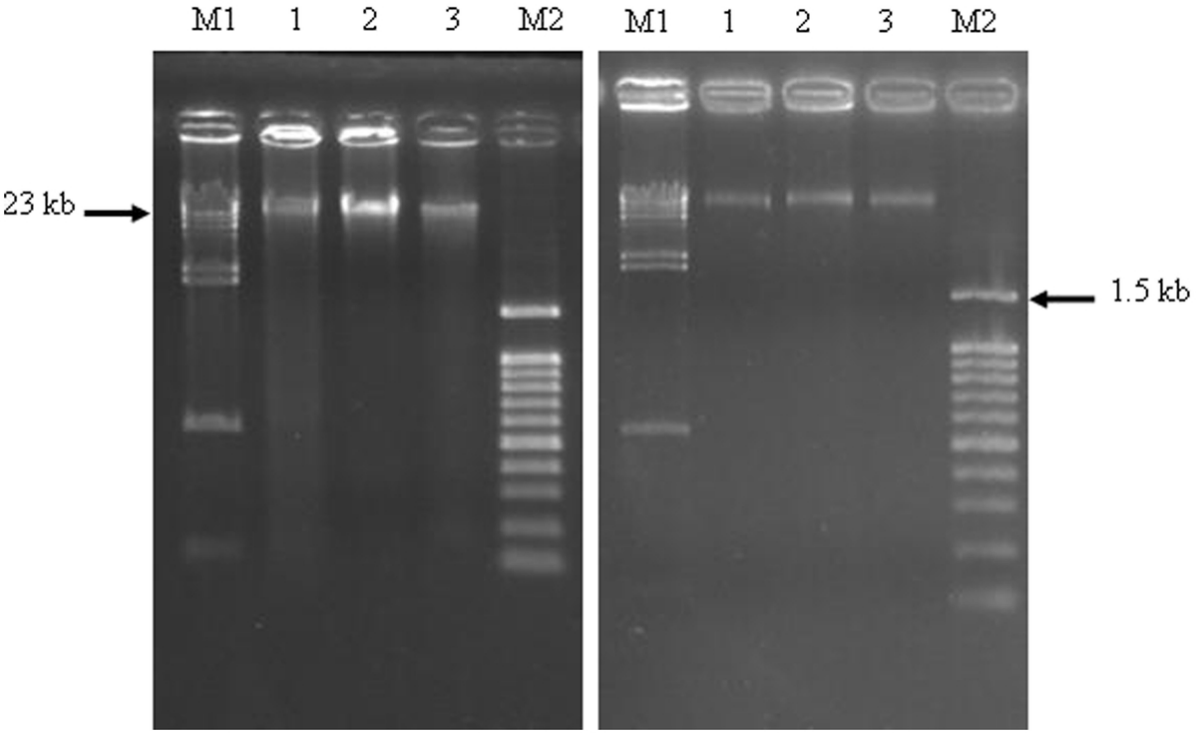
**Agarose (1.8% (w/v))-TBE gel electrophoresis of 1 μg DNA (per lane) extracted from (A) SW620 and (B) Hs27 cells after 72 h *in vitro *culture in complete medium supplemented with (Lane 1) DMSO solvent only (control), (Lane 2) compound 1 (cardanol) at its IC_50 _value (10.76 μg/ml) and (Lane 3) compound 2 (cardol) at its IC_50 _value (3.0 μg/ml)**. Lanes M1 and M2 contain λ *Hin*d III and 100 bp ladders, respectively, as DNA size markers.

In order to find out whether compounds 1 and 2 (cardanol and cardol) could induce apoptosis or necrosis through damage to the DNA of the cells in culture or not, the DNA was extracted from cultured SW620 cells and examined for size following resolution by agarose-TBE gel electrophoresis. If they play no role in DNA damage, then the DNA would be expected to be intact and appear as a high molecular weight and sharp band following agarose - TBE electrophoresis, whereas, in contrast, if significant damage to the DNA was induced then a smear of fragmented DNA or a 180-200 bp interval ladder (apoptosis) will be seen. Neither compound 1 (cardanol) nor compound 2 (cardol) treated SW620 cells or the Hs27 cells revealed any evidence of fragmentation of the DNA, neither as an apoptotic ladder nor a general degradation smear (Figure 8).

From the analysis of the extracted DNA, which was a large single band and not a 180-200 bp ladder or smear, it is possible that compounds 1 and 2 did not kill the cells by apoptosis since no DNA ladder pattern was seen. In addition, no smear was found suggesting no significant level of DNA damage. This does not contrast with the notion of death by necrosis, as suggested by the morphology changes, since the badly damaged (necrotic) cells would have been removed in the washing process during cell harvesting and before DNA extraction.

## Discussion

In this research, propolis from *A. mellifera *was used to determine the *in vitro *antiproliferative/cytotoxic activity on five human cancer cell lines. Although there are many bee species that can produce propolis, especially stingless bees, such as *Melipona fasciculate*: [30] and *Tetragonula carbonaria *[31], *A. mellifera *was chosen since it is commonly cultured for honey, is an easy to manage species in apiaries and so makes access to propolis on a commercial, as well as environmentally sustainable, scale feasible. In addition, the bioactivities of propolis are reported to depend on the geographical regions [32], seasons [14] and other external factors. Thus, the propolis of *A. mellifera *from Thailand, a floral biodiversity hotspot, is of interest since it has never been reported previously yet maybe different from the propolis of this species reported previously from other regions. The selection of Nan province was based upon the diverse flora still present in this region of Thailand, and so the potential for novel compounds in the propolis. This native and remote area of the country is dry, mountainous and full of deep forests with unique plants, such as *Bretschneidera sinensis *Hemsl.

Propolis was initially sequentially extracted with MeOH (high-polar solvent), then CH_2_Cl_2 _(medium-polar solvent) and finally hexane (non-polar solvent). Both the hexane (CHE) and CH_2_Cl_2 _(CDE) extracts revealed a good antiproliferative/cytotoxic activity against the five selected human cancer cell lines, as determined by the MTT assay. Thus, in general the antiproliferative/cytotoxic compounds in this propolis from *A. mellifera *in Nan, Thailand, are unlikely to be highly polar. This notion is supported by Castro et al. [33] who reported the best antiproliferative activity against HeLa tumor cells was from prenylated benzophenone (hyperibone A), which is found in the CHE of Brazilian propolis, with an IC_50 _value of 175.6 nM (91 ng/ml).

Both MeOH and water/EtOH, two polar solvents, could be used to extract the antioxidant activity from propolis from Portugal [34], whilst other optimal extraction solvents were reported to be chloroform for the antimicrobial activity against oral pathogens [30] and ethanol for the anti-influenza A virus activity [35]. Thus, the bioactivities of crude propolis extracts, and so the frequently, albeit incorrectly, inferred propolis bioactivities, depend also on the extraction solvents used as well.

The different cell line sensitivities and IC_50 _values for the antiproliferative/cytotoxic activity before and after fractionation by adsorption chromatography could represent the removal of inhibitory components that exert an antagonistic effect, or the separation of different components with different activities. Comparing the IC_50 _values of compounds 1 and 2 (Table 5 and Figure 3), compound 2 (cardol) looked to be a promising agent for anti-cancer treatment in terms of its lower IC_50 _values for antiproliferation/cytotoxicity compared to compound 1 (cardanol), assuming that (i) the same IC_50 _values observed against the non-transformed Hs27 cell line reflects an antiproliferative activity only and not a cytotoxic activity and that (ii) a specific delivery system could be used to target the cancer cells or tumor area rather than systemic delivery, so as to avoid or minimize side affects. Moreover, consumption of the crude form of propolis should be warned against because Aliboni et al. [26] reported that propolis can cause an allergic reaction to sensitive individuals due to the presence of the two allergenic esters, benzyl salicylate and benzyl cinnamate.

Both compounds 1 and 2 (cardanol and cardol) are phenolic lipids with an amphiphilic character [36] derived from the hydrophilic hydroxyl group and the hydrophobic long chain hydrocarbon [37]. These compounds are found in tropical plants in the family Anacardiaceae, both in native and cultivated cultures [38]. Economic plants in this family include cashew nut, mango and ginkgo [39], whilst the diversity of both compounds is high, such as in the form of anacardic acid, catechol, resorcinol and gingkolic acid [37]. Indeed, members of these groups have previously been reported to exhibit diverse bioactivities, such as antibacterial [40], antiplasmodial [41], antioxidant [42] and antifungal activities [43]. However, the diversity of chemical structures in the cardanol and cardol groups may account for the diverse bioactivities [44], rather than a few pluripotent compounds.

Wang et al. [45] reported that they could purify CAPE from propolis, and that it showed an antiproliferative activity on the human colorectal cancer cell line (CRC) in a dose- and time-dependent manner. The IC_50 _value of CAPE after 72 h treatment was 22.7 μM (6.47 μg/ml). Comparing compound 2 (cardol) from our research with that for CAPE, the antiproliferative/cytotoxic activity IC_50 _value of compound 2 on the SW620 cell line (< 3.13 μg/ml; < 6.8 μM), which is also a human colorectal cancer cell line, was over 3.3-fold lower than the IC_50 _value of CAPE on CRC (in terms of molarity). Thus, subject to the risk of side effects, compound 2 (cardol) purified from Thai *A. mellifera *propolis could be a better antiproliferative agent against human colorectal cancer cells.

CAPE is also reported to have an effect on breast cancer cells, with a similar IC_50 _value on the ER^- ^and ER^+ ^MDA-231 and MCF-7 cell lines, respectively, of 15 μM (4.26 μg/ml) [22]. Thus, the IC_50 _value reported for CAPE is broadly similar in terms of mass, but some 1.5-fold higher in terms of molarity, to that seen here for compound 2 (cardol) against the breast cancer cell line BT474 (4.41 μg/ml; 9.61 μM), again indicating that cardol purified from Thai *A. mellifera *propolis could be an interesting antiproliferative agent against human breast cancer cells.

CAPE has been reported to display a broad target range inhibiting the growth of many cancer cell lines, such as C6 glioma cells [46] and human leukemia (HL-60) cells [47], and also to be cytotoxic to the neck metastasis of gingiva carcinoma (GNM) and tongue squamous cell carcinoma (TSCCa) cells [48]. Moreover, CAPE showed a strong inhibitory effect on the matrix metalloproteinase (MMP-9), which is related to the invasion and metastasis ability of hepatocellular carcinomas [49]. In the future, the effect of compounds 1 (cardanol) and 2 (cardol) from this Thai *A. mellifera *propolis should be evaluated accordingly.

Since many cancer drugs or chemotherapy agents used nowadays cause adverse side effects through being cytotoxic to normal cells, it is necessary to find new compounds that will not cause such adverse side effects and not be cytotoxic to normal cells. Therefore, the apparent absence of cytotoxicity of compounds 1 (cardanol) and 2 (cardol) to the non-transformed Hs27 cell line *in vitro *is of interest, but requires conformation in a broader range of non-transformed cell lines. However, against that was the observed antiproliferative affect noted on the Hs27 cell line, which may well then result in strong adverse side affects and so the requirement for more localized drug delivery systems. This is because although compounds 1 (cardanol) and 2 (cardol) affected some cancer cell lines *in vitro *with lower IC_50 _values than that against the non-transformed Hs27 cell line, this small difference is unlikely to be sufficient to allow safe systemic administration without side affects, but may be sufficient when targeted local delivery is performed [50,51].

Propolis and its phenolic compounds have been reported to induce the death of cancer cells either by necrosis [52] or by apoptosis, the latter of which might be by mitochondria mediated- [21] or death signal mediated- [53] apoptosis. Thus, the *in vitro *effects of compounds 1 and 2 upon the cell morphology and DNA fragmentation of the cell lines was observed.

A change in the cell morphology with a decrease in the cell number was observed for SW620 cells when cultured *in vitro *with compounds 1 (cardanol) or 2 (cardol), which is consistent with a cytotoxic effect. In contrast, no change in the cell morphology was observed with the Hs27 cells under the same conditions. It is likely that compounds 1 (cardanol) and 2 (cardol) affected the SW620 cancer cells by necrosis, not by apoptosis, whereas they induced an antiproliferation response and not cell death in the Hs27 cells. In contrast, Vatansever et al. [54] reported that CEE from Turkey induced the death of the human breast cancer cell line (MCF-7) by the induction of apoptosis. Although the morphology of the MCF-7 cells was not visibly changed, the number of cells was decreased. In addition, whilst Umthong et al. [55] found that CWE and CME from *Trigona laeviceps *(stingless bee) in Samut Songkram province, Thailand, had a similar effect upon SW620 cells as that reported here (change in the cell morphology, loss of cell adhesion and cell death), in contrast, they found evidence of DNA fragmentation, unlike in this study with compounds 1 (cardanol) or 2 (cardol). Moreover, Chen et al. [56] reported that propolins A and B extracted from Taiwanese propolis could induce apoptosis of human melanoma A2058 cells, in addition to inducing the morphological changes in the cells, chromatin condensation and cell shrinkage. However, since we did not screen the crude extracts for changes in the cell morphology and DNA damage, but only the two purified compounds that were not propolin A or B, then it is unclear if this represents the diversity of bioactivity within different propolis components or between propolis samples.

Cancer can be caused by the misregulation of, and so its treatment can be targeted at inhibition of, phosphatidylinositol-specific phospholipase Cγ1 (PI-PLCγ1), since it plays a key role in the proliferation and progression of human cancer [57]. Thus, an inhibitor of PI-PLCγ1 would be a useful tool for development of anticancer agents. Lee et al. [58] reported the isolation of a cardanol from the chloroform extract of *Ginko biloba *that exhibited inhibitory effects against PI-PLCγ1 in a concentration-dependent manner. They also found that the structure of the cardanol could influence the inhibitory effect. Cardanol with unsaturated long carbon chains (cardanol C_15:1 _and cardanol C_17:1_) showed more potent activities than those with saturated long chains (cardanol C_13:0 _and cardanol C_15:0_). Other than the inhibition on PI-PLCγ1, cardanol is reported to be cytotoxic *in vitro *to human cancer cell lines, such as HCT-15 (colon), MCF-7 (breast), A-549 (lung), HT-1197 (bladder) and SKOV-3 (ovary), but was not found to be cytotoxic to the normal colon cell line, CCD-18-Co.

In addition, Kubo et al. [59] reported that the cardol (C_15:0_) isolated from *Anacardium occidentale *was moderately cytotoxic to the murine B16-F10 melanoma cells in a dose-dependent manner with an IC_50 _value of 24 μM (8.352 μg/ml) and complete lethality at 40 μM (13.92 μg/ml), which in terms of molarity is some two- to 3.5- fold higher than that observed here for compound 2 (cardol) from the Thai *A. mellifera *propolis (albeit subject to the caveat of on different cell lines). Since cardol is an amphipathic molecule, the cytotoxicity is potentially facilitated by its ability to act as a surfactant.

The two potentially new compounds isolated here from Thai *A. mellifera *propolis (a cardanol and a cardol) could be alternative antiproliferative agents for future development as anti-cancer drugs.

## Conclusion

Propolis of *A. mellifera *was focused upon in this research due to the wide cultivated distribution of this bee species in Thailand, a floral biodiversity hotspot. The location of Nan province was accordingly selected due to the native and remote area of the country. Since the crude hexane and dichloromethane extracts of propolis provided a good *in vitro *antiproliferation/cytotoxicity against the selected cancer cell lines, it indicated that the polarity of the active compounds is likely to be low. Considering the cell line sensitivities and IC_50 _values for the antiproliferation/cytotoxicity before and after each fractionation, application of the active crude extracts is more interesting. After purification and chemical structure analysis, one member of each of the cardanol and cardol groups, as phenolic compounds, were revealed. The apparent absence of cytotoxicity of both compounds to the normal Hs27 cell line *in vitro *is of interest since many cancer drugs or chemotherapy agents used nowadays cause adverse side effects through being cytotoxic to normal cells. Considering the cell morphology, cell number and the cytotoxic effect, it is likely that both compounds affected the SW620 cancer cells by necrosis.

## Competing interests

The authors declare that they have no competing interests.

## Authors' contributions

DT performed the experiments. PP contributed to the study design, analyzed and interpreted the data. SP contributed to the invaluable help in cell culture. KK, MO, HM and AK contributed to invaluable suggestions and comments on research. CC did the design and supervision of the experiments. Also, she contributed in drafting the manuscript. All authors read the manuscript, contributed in correcting it and approved its final version.

## Pre-publication history

The pre-publication history for this paper can be accessed here:

http://www.biomedcentral.com/1472-6882/12/27/prepub
